# Identifying Ingredient Substitutions Using a Knowledge Graph of Food

**DOI:** 10.3389/frai.2020.621766

**Published:** 2021-01-25

**Authors:** Sola S. Shirai, Oshani Seneviratne, Minor E. Gordon, Ching-Hua Chen, Deborah L. McGuinness

**Affiliations:** ^1^ Rensselaer Polytechnic Institute, Troy, NY, United States; ^2^ IBM T. J. Watson Research Center, Yorktown Heights, NY, United States

**Keywords:** ingredient substitution, semantic similarity, semantic substitution, knowledge graph, patient empowerment, health empowerment

## Abstract

People can affect change in their eating patterns by substituting ingredients in recipes. Such substitutions may be motivated by specific goals, like modifying the intake of a specific nutrient or avoiding a particular category of ingredients. Determining how to modify a recipe can be difficult because people need to 1) identify which ingredients can act as valid replacements for the original and 2) figure out whether the substitution is “good” for their particular context, which may consider factors such as allergies, nutritional contents of individual ingredients, and other dietary restrictions. We propose an approach to leverage both explicit semantic information about ingredients, encapsulated in a knowledge graph of food, and implicit semantics, captured through word embeddings, to develop a substitutability heuristic to rank plausible substitute options automatically. Our proposed system also helps determine which ingredient substitution options are “healthy” using nutritional information and food classification constraints. We evaluate our substitutability heuristic, diet-improvement ingredient substitutability heuristic (DIISH), using a dataset of ground-truth substitutions scraped from ingredient substitution guides and user reviews of recipes, demonstrating that our approach can help reduce the human effort required to make recipes more suitable for specific dietary needs.

## 1 Introduction

Eating habits can play an important role in improving personal health. For example, dietitians recommend that patients diagnosed with diabetes monitor their intake of specific nutrients from foods (like carbohydrates and protein) to treat their condition (American Diabetes Association, 2020). If people need to adjust their intake of some nutrients, they may choose to modify their diet by substituting ingredients in their meals. Such substitutions can remove restricted types of ingredients (e.g., common allergens) or replace ingredients to adhere to some dietary constraint (e.g., replacing potatoes to reduce carbohydrate intake). Substituting individual ingredients rather than strictly following a new meal plan allows people to eat familiar meals while maintaining their dietary goals.

People who are looking to make substitutions have several common resources available. Popular recipe websites, such as AllRecipes^1^ and Food.com^2^, have comment sections where community members discuss their opinions and modifications of different recipes. These websites also compile lists of common ingredient substitutions^3^. Another approach is to use popular search engines. Queries to search engines, using keywords such as “potato substitute,” return relevant results from a variety of websites, and queries targeting specific nutrients (e.g., “low-carb substitutes”) can sometimes find websites aggregating common substitution options for such diets.

But to make use of such basic search methods, people require knowledge about how specific ingredients impact their overall health. They also must spend extra effort to determine if potential substitutions are “healthier” or somehow “better” in the context of their dietary needs. For example, if someone wants to find gluten-free substitutions for all-purpose flour substitutions and has a goal to reduce carbohydrate intake, search results might focus solely on avoiding gluten. They must perform additional searches to identify which substitutions also contain fewer carbohydrates than all-purpose flour. Searching for substitutions for less common ingredients may also be difficult, especially if a person has additional dietary restrictions that they wish to apply. This added effort makes it difficult for people to make recipe modifications tailored to their nutritional needs.

Efforts to apply machine learning methods to automatically select substitutions have been limited due to the lack of any widely accepted dataset of valid substitutions. Other existing works, such as the study by Gaillard et al. (2014), define explicit rules for substitutions, but such rules are typically not scalable because they are only defined for a narrow set of recipes and rely on detailed annotations about recipes and ingredients. Existing works also do not consider examining automatic ingredient substitution together with constraints on nutritional information.

Our work can empower people to more effectively modify their eating habits by simplifying the process of identifying plausible ingredient substitutions and comparing ingredients based on their food classification and nutritional content. While examples in this study are mainly aimed at improving diets for people concerned with preventing or managing diabetes, the work can be used for a wide range of food substitution tasks. We facilitate this using our team’s FoodKG (Haussmann et al., 2019), a knowledge graph of recipe and ingredient information. FoodKG uses linked data to capture an ingredient’s food categorization, from FoodOn (Dooley et al., 2018), and nutritional information, from the USDA^4^.

We use linked semantic information about ingredients to develop a substitutability heuristic for automatically ranking plausible ingredient substitutions. We also use the linked nutrition and classification to identify ingredients in the recipe and potential substitutions that should be modified to adhere to specific dietary constraints. Together with the vast catalog of recipes in FoodKG, these functionalies will enable people to more easily identify ingredient substitutions suited to their diet.

### 1.1 Contributions

This study presents an approach to navigate the search space of valid ingredient substitutions in a recipe to satisfy dietary constraints and rank plausible substitutions. Our contributions are as follows:

- Develop a novel ingredient substitutability heuristic, DIISH, which leverages explicit semantic information and word embeddings of ingredients to rank plausible substitutions (Section 2.3)
- Evaluate our substitution ranking heuristic using ground-truth substitutions collected from web resources and user reviews of recipes (Section 2.4)
- Provide a design and demonstration implementation that uses linked nutritional information and food classifications to determine “healthy” ingredient substitution options that satisfy personal dietary constraints (Section 2.2)

## 2 Methods

### 2.1 Ingredient Substitution in Recipes

Recipes in their most basic form consist of a list of ingredients, the quantities in which they are used, and several instructions to form the final dish. Individual ingredients can serve various purposes within recipes depending on the preparation steps and other ingredients in the recipe.

Of the many motivations for ingredient substitution, we focus on two representative cases. The first is to satisfy dietary restrictions on specific types of ingredients, such as replacing meat-based ingredients for vegetarian diets or replacing allergens such as peanuts. The second is to modify the nutritional contents of meals (e.g., replacing high-carb ingredients with low-carb alternatives).

Whether a substitution is “good” can be highly subjective. Arguably, good substitutions have the goal of replacing an ingredient to improve some criteria while maintaining the essence of the original recipe (while intentionally creating new dishes may be a valid goal, it is not in the scope of this work). Some criteria to consider for this goal include the flavor profile (e.g., sweet or savory) and texture (e.g., crunchy or chewy) of ingredients. Substitutions may also need to consider fulfilling some functional role in the recipe, such as how eggs act as emulsifiers in some sauces and custards. Such aspects of ingredients and their roles in a recipe are seldom explicitly captured, making it difficult to determine how good a substitution is at replicating the original ingredient in all dimensions.

While the quality of an ingredient substitution can be subjective and difficult to concretely determine, we can objectively judge whether substitutions are good in terms of satisfying constraints. For example, substitutions aiming to reduce the amount of carbohydrates in a dish are objectively bad if the substitute ingredient contains more carbohydrates than the original. As another example, suggesting meat-based substitution for a vegetarian is objectively bad.

One might begin by comparing the similarity metrics between ingredients to judge their substitutability to find potential ingredient substitutions. For example, some indications that one ingredient would be a good substitute for another might be that they have similar flavors (e.g., sweet, sour), similar textures (e.g., crunchy, fluffy), similar food categorizations (e.g., substituting different varieties of potatoes), or are used in similar recipe contexts (e.g., using bacon or chicken as the main protein in a sandwich).

However, challenges exist in using each of these possible sources of substitution information. Properties of ingredients such as flavor molecules and compounds have been captured in resources such as FlavorDB (Garg et al., 2018) or FooDB (The Metabolism Innovation Center, 2017), but it is difficult to directly relate these chemical features to the experience of flavor in recipes. The use of food categorization similarity is a plausible method to find simple substitutions (like different varieties of potatoes). However, this method will struggle to discover good substitutions that are not closely related (e.g., a vegan substitution for meat will have vastly different food categorization). Finally, the similarity in recipe contexts can become a struggle when the space of the recipe contexts is large but sparse.

The data source for recipes and ingredients used in this study, FoodKG, captures links from ingredients to nutritional information and ontology of food. The information captured in FoodKG is well suited for our motivation of substitutions because it enables a detailed analysis of nutritional intake and the categorization of individual ingredients. It also encapsulates a very wide variety of ingredients and cuisines (over 17,000 ingredients and over one million recipes). To overcome the limitations of purely semantics-based approaches, we employ external sources of latent semantic information to capture further ingredient properties.

### 2.2 Identifying Target Ingredients and Healthy Substitutions

**FIGURE 1:**
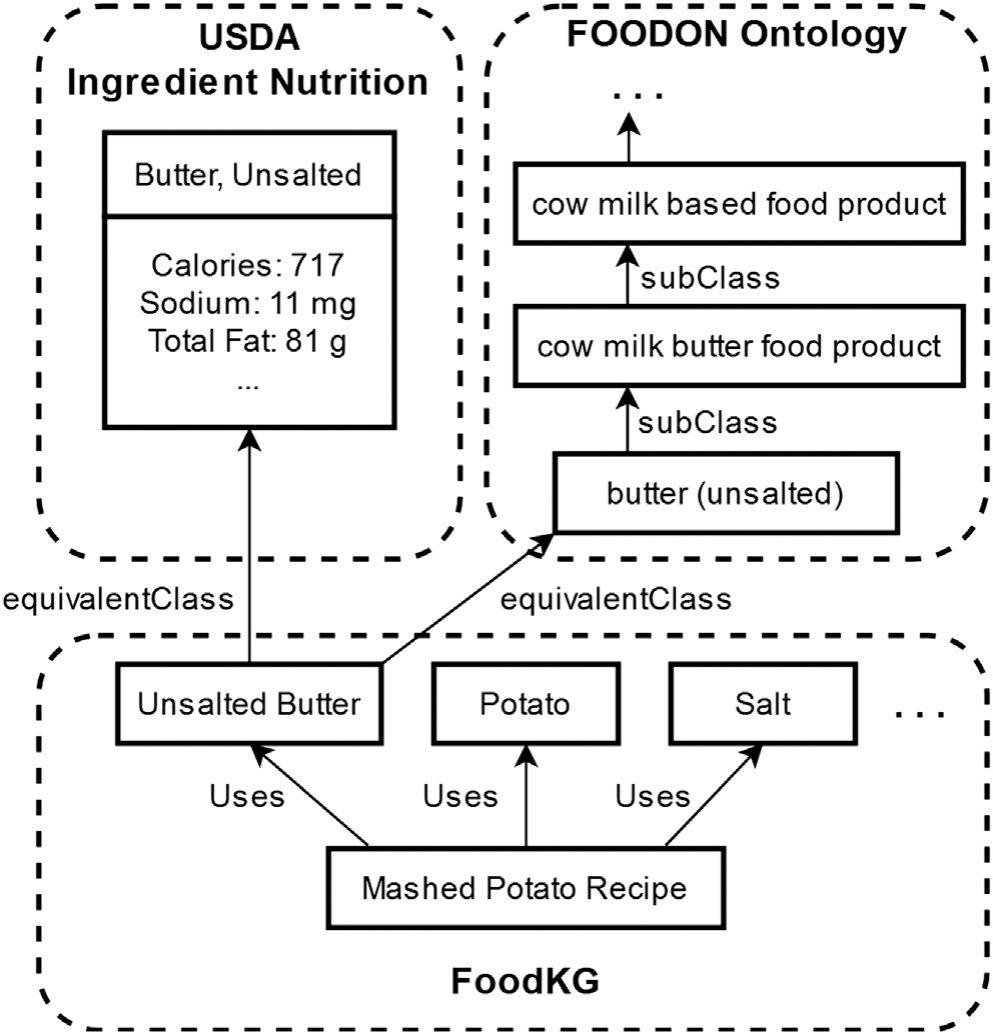
Example of linked ingredient information in FoodKG for the ingredient “Unsalted Butter” to the matching class in FoodOn’s ontology and the USDA’s nutritional information.

The key use case that motivates our work is to perform ingredient substitutions in recipes that will satisfy personal dietary constraints. The two categories of dietary constraints that we consider in this work are restrictions on the types of ingredients that may be consumed and limitations on the consumption of certain nutrients. Based on these constraints, in order to produce “healthier” substitutions for ingredients, we must identify which “unhealthy” ingredients to remove from a recipe as well as which substitution options are “healthy.” Here, we use linked ingredient information present in FoodKG to compare nutritional information and food categorization to determine whether they are “healthy” for a particular dietary context. Figure 1 shows an example of how an ingredient in FoodKG is linked to entities in the FoodOn ontology and the USDA’s nutrition information.

For restrictions on types of food, we use FoodOn classes to identify ingredients that violate the restriction. For each ingredient contained in the target recipe, we retrieve its linked FoodOn class and identify whether there exists a path in the ontology hierarchy (through relationships such as “subClassOf” and “derivesFrom”) between the prohibited food class and the ingredient. Similarly, we identify options for healthy substitutions by filtering out ingredients that are subclasses of prohibited foods.

For the case of restrictions over the nutritional content of recipes, we must calculate the nutritional content of each ingredient the recipe uses. Ingredients in FoodKG are linked to the USDA’s food data, which presents nutritional information (such as grams of carbohydrates or milligrams of sodium) per 100 g of each ingredient.

To convert ingredient measurements into grams, we use text descriptions for common units of measurement presented by the USDA (e.g., the entry for “BUTTER, WITH SALT” has the common units “1 pat” and “1 tbsp” as well as their corresponding gram weights). We used these descriptions to convert ingredient measurements in FoodKG into grams, using the first available common units as the default weight. Where possible, nonstandard units (e.g., “1 can of beans”) were matched against the ingredient’s common unit descriptions.

To assess the correctness of our nutrition calculation method, we applied it to calculate the calorie information for a set of 8,659 recipes whose ingredients all had links to USDA foods in the FoodKG. Compared to the ground-truth calorie information (scraped from Food.com) for this set of recipes, the average per serving error of our calculated nutrition was 34.51 calories. Our error is comparable to a similar method demonstrated by Kalra et al. (2020), who also used the USDA’s data to calculate calorie information for a set of 2,482 recipes and reported an average per-serving error of 36.42 calories. To put this into perspective, 1 ounce of chicken breast contains roughly 47 calories. If we consider that many unclear measurements, such as “1 chicken breast,” may vary in weight by several ounces, this error seems within a reasonable range.

Once this nutritional information is calculated, total nutritional information for recipes can be analyzed to determine whether they violate personal dietary goals. If a recipe is found to violate the dietary goals, ingredients that contribute the most to a particular nutrient that the person is limiting can be identified as candidate ingredients to substitute out of the recipe. We use a similar approach to select substitutions that are “healthier” for the user by comparing nutritional information to the target ingredient. If someone has a goal to reduce carbohydrates, for example, then we only should suggest substitute ingredients containing fewer carbohydrates than the original ingredient.

### 2.3 Ranking Plausible Substitutions

Once we have determined which options for substitutions are considered “healthy,” we must turn our attention to determining how to rank the substitutions.

Intuitively, our approach to ingredient substitution ranking should consider similarities in the properties of ingredients, other ingredients they are frequently used together with, and the recipes in which they are used. We also must consider that some senses of “similarity” may not directly connect to substitutability. For example, ingredients that are used together frequently (e.g., garlic and olive oil) may be similar but not substitutable. Conversely, ingredients that are dissimilar in terms of food classification may be good substitutions (e.g., potatoes and cauliflower). In order to select good options for substitute ingredients, we develop a heuristic incorporating latent and explicit semantics about ingredients.

We use two sources of latent semantic information about ingredients as word embeddings based on ingredient names. The first is a Word2Vec (Mikolov et al., 2013) model trained over ingredient names and recipe instructions from Recipe1M (Marin et al., 2019). The second is the spaCy’s word embedding model (Honnibal et al., 2017). We use these two models hoping to capture latent semantics specific to FoodKG’s data as well as more general (or at least not food-focused) language. We use each word embedding model to compute the cosine similarity between ingredient names separately. For ingredients *a* and *b*, the cosine similarity of their Word2Vec and spaCy embeddings are represented as $W_{a,b}$ and $S_{a,b}$, respectively.

As for the explicit semantic information from FoodKG, we compute two additional scores to help rank substitution options.

**FIGURE 2:**
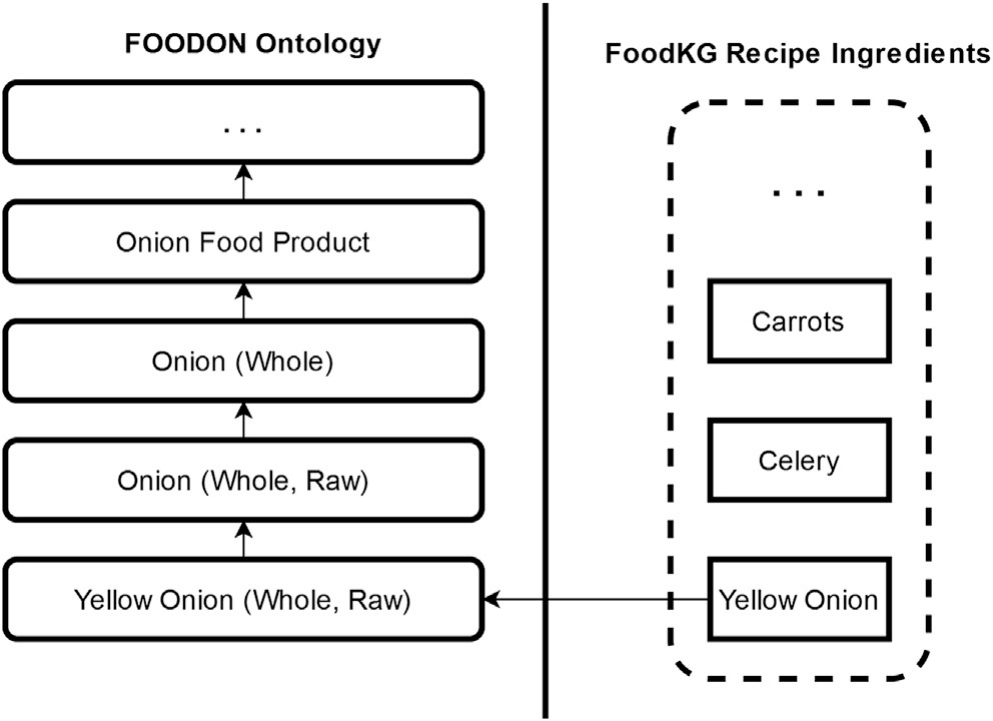
An example of the linkages between ingredients in FoodKG and FoodOn’s ontology. Subclass relations of FoodOn ingredients are used to enhance our scoring metrics.

The first score builds on the intuition that a good substitution ingredient should pair well with the recipe’s other ingredients. To capture this intuition, we compare the similarity of ingredients that co-occur in recipes alongside the target ingredient. We also leverage the links to FoodOn’s food class hierarchy for each ingredient to make this heuristic more generalized. Thus, for example, rather than only looking at how frequently ingredients *a* and *b* were used with “yellow onions,” we can consider the frequency of use of ingredients that are linked to “onion (whole, raw),” “onion (whole),” and so on, as shown in Figure 2.

For ingredients *i* and *j* in the set of all ingredients *I*, let $T_{j,i}$ represent the number of recipes in which *j* is used either with ingredient *i* or an ingredient that is a subclass of *i*. Computing this for all ingredients, as $\sum_{i\in I}T_{j,i}$, can give us a vector representing the probability of each ingredients co-occurring in a recipe together with ingredient *j*. We can then compute the co-occurrence substitutability score $D_{a,b}$ between ingredients *a* and *b* as the cosine similarity between these vectors.

$$D_{a,b}=\frac{\sum_{i\in I}T_{a,i}T_{b,i}}{\sqrt{\sum_{i\in I}T_{a,i}^{2}}\sqrt{\sum_{i\in I}T_{b,i}^{2}}}.$$

We additionally wish to capture the notion of ingredients being used in similar recipe contexts. Following the work of Achananuparp and Weber (2016), for each ingredient and recipe context, we compute the positive pointwise mutual information (PPMI). Here, the recipe context refers to the other ingredients used in a recipe. For example, in a recipe consisting of (bread, peanut butter, and jelly), jelly is used in the context (bread and peanut butter).

We once again use FoodOn links to better generalize the recipes by converting ingredients to their equivalent FoodOn classes. This conversion helps reduce the space of ingredients that we are considering, as FoodKG has many cases of ingredients that link to the same FoodOn class (e.g., “tomatoes,” “ripe tomatoes,” and “diced tomatoes” all link to a tomato class in FoodOn). We also consider superclass relations to generalize the occurrence of ingredients within recipe contexts. For a given ingredient *i* that occurs with recipe context *c*, we also consider any superclass of *i* to be occurring with *c*.

Let $F_{i}$ be the number of times ingredient *i* occurs in FoodKG, $F_{c}$ be the number of times the context *c* occurs, and $F_{i,c}$ be the number of times *i* is used with the context *c*. We then calculate PPMI as

$${\text{PPMI}}_{i,c}=\max\left(\log\frac{F_{i,c}}{F_{i}*F_{c}}*\sqrt{\max\left(F_{i},F_{c}\right)}\right).$$

For each ingredient, we form a vector of the computed PPMI between that ingredient and context *c* in the set of all contexts *C*. For ingredients *a* and *b*, we compute the score $P_{a,b}$ as the cosine similarity between these vectors. Thus, PPMI gives this metric some indications of how ingredients relate to recipes, and $P_{a,b}$ then captures the similarity between how two ingredients relate to recipes.

$$P_{a,b}=\frac{\sum_{c\in C}{PPMI}_{a,c}{PPMI}_{b,c}}{\sqrt{\sum_{c\in C}{PPMI}_{a,c}^{2}}\sqrt{\sum_{c\in C}{PPMI}_{b,c}^{2}}}.$$

#### 2.3.1 Diet-Improvement Ingredient Substitutability Heuristic

We create our final heuristic, DIISH, by combining the four previously mentioned scores. To combine the scores, we used a randomly selected set of 100 target ingredients from our evaluation data (described in Section 2.4.1) to serve as a development dataset. Using this development set, we explored choices for coefficients and powers to apply to each metric in order to form our final heuristic.

Coefficients were selected from [0.5,1,2,4], and the powers were selected from [1/4,1/2,1,2]. Since each metric has an upper bound of 1, taking the square root would generally increase the influence of the metric, while squaring it would decrease its influence. We left our selection of coefficients and powers relatively simple, as the focus of our work was to demonstrate the usefulness of combining multiple metrics rather than highly optimizing the combination to our data.

All combinations of our selected set of coefficients and powers were applied to form roughly 65,000 different combinations of the four scoring metrics. We evaluated the performance of each combination using our development dataset and selected the best combination to form our final heuristic. We calculate the DIISH score of two ingredients as

$${DIISH}_{a,b}=W_{a,b}+S_{a,b}^{2}+\frac{1}{2}\sqrt[{4}]{D_{a,b}}+2\sqrt{P_{a,b}}.$$

#### 2.3.2 Filtering

As our last strategy for identifying substitutions, we leverage the FoodOn subclass information to rule out options that would likely fail to serve as substitutes. For example, if we have a general ingredient, such as potatoes, it would make very little sense to suggest a specific variety of potato as an option for substitution and vice versa. We thus filter out options for substitutions that are super or subclasses of the target ingredient.

### 2.4 Evaluation

Evaluating the quality of ingredient substitutions is difficult due to the lack of any widely accepted ground-truth set of valid substitutions; one’s opinion on the validity of an ingredient substitution may be influenced by personal preference and perceived purpose of the substitution or their culinary experience. Other works typically perform user studies for evaluation (Achananuparp and Weber, 2016; Akkoyunlu et al., 2017).

In the absence of an existing ground-truth for this task, we opted to collect a few new sets of ingredient substitutions from selected web resources to use as a ground-truth to compare our results. While this choice cannot fully overcome the subjectivity challenges, we believe the data source we drew from will provide a reasonable approximation of human judgment while covering a scope of substitutions of which many home-cooks may not be aware.

#### 2.4.1 Substitution Dataset Collection

For this evaluation, we only consider substitutions where a single ingredient replaces another. We also allow for a single source ingredient to have many options for substitutions (e.g., the target ingredient potato may have substitutes of cauliflower, rutabaga, and carrots). We also do not assume substitutions to be reflexive (i.e., cauliflower being a substitute for potatoes does not imply potatoes are a substitute for cauliflower).

We collected three sets of data to serve as ground-truth ingredient substitutions to compare our results against. The first was a relatively small set of manually curated substitutions from “common ingredient substitution” guides published by AllRecipes^5^, Food Network^6^, Colorado State University^7^ (CSU), and North Dakota State University^8^ (NDSU). The second was scraped from The Cook’s Thesaurus^9^, which contains a variety of information about ingredients as well as common substitutes. Last, we extracted substitutions from 1.1 million user reviews of Food.com recipes published by Majumder et al. (2019). We parsed these user reviews using simple patterns indicating that the user made a substitution to the recipe (e.g., “substitute *a* for *b*” or “replace *b* with *a*”). Table 1 shows the number of substitution pairs (i.e., pairs of “target ingredient” to “substitute ingredient”) and unique ingredients present in each dataset.

**TABLE 1:** A breakdown of evaluation data sources and substitutions.

Source	# substitution pairs	# unique ingredients
AllRecipes	137	72
Food Network		
CSU		
NDSU		
The Cook’s Thesaurus	2,360	1,004
Majumder et al.	3,313	1,331

#### 2.4.2 Poincaré Embeddings

To compare the performance of our method against other recent works in the domain of food, we additionally develop a Poincaré embedding model (Nickel and Kiela, 2017) using FoodOn’s class hierarchy. FoodEx2Vec (Eftimov et al., 2020) demonstrated the utility of Poincaré graph embeddings, which can capture hierarchical relations between terms, to develop embedding for a food classification system. As the embeddings of FoodEx2Vec cannot be directly applied to our foods, we chose to follow the same methodology using FoodOn to generate a new model to compare our results against.

We apply the methods presented by Eftimov et al. (2020), using the implementation available from Gensim (Řehůřek and Sojka, 2010). Subclass relations from FoodOn are used to train the model for 50 epochs, and we produce 50-dimensional embeddings for ingredients. To compare our methods against the Poincaré embedding model, we compute the cosine similarity between ingredient vectors to rank substitution options.

## 3 Results

### 3.1 Example Substitutions

Table 2 displays two examples of substitution options produced by our approach that show good performance against our ground-truth dataset. The ground-truth substitution options do not have any ranked order, while our approach here presents the top five ranked results. For arugula, we can see that watercress and radicchio are first and third highest ranked substitutions, respectively, and the other ingredients in the top five options also appear to be reasonable suggestions for leafy vegetables. For lard, four of the top five ranked ingredients matched with those from the ground-truth data. “Shortening” is also commonly understood to mean shortening made from vegetable oil, so it may also be appropriate to consider the top-ranked option of vegetable shortening to be correct.

**TABLE 2:** Examples of ground-truth substitutions compared to substitute options ranked by our approach.

Target ingredient	Ground-truth substitutes	DIISH’s top 5 ranked substitutes
Arugula	Watercress	Watercress
	Belgian endive	Frisee
	Radicchio	Radicchio
	Escarole	Romain lettuce
		Butter lettuce
Lard	Vegetable oil	Vegetable shortening
	Shortening	Shortening
	Margarine	Margarine
	Bacon fat	Bacon fat
	Butter	Butter

### 3.2 Ingredient Substitution Ranking Results

We evaluate the quality of our substitutions based on rankings of substitute ingredients using their computed substitutability score. For each dataset of substitutions, we only consider the set of unique ingredients present in that dataset for substitute ranking.

For each target ingredient in our ground-truth, we computed all other ingredients’ substitutability scores to produce a ranked list of substitution options. We also applied our filtering strategy to all scoring metrics.

We frame our approach as an information retrieval problem and evaluate our results using mean average precision (MAP), mean reciprocal rank (MRR), and recall rate at k (RR@k). Recall rate at *k* is calculated as the proportion of target ingredients for which any correct substitute option falls within the top *k* ranks. We calculated the MAP and MRR using the ranking results of all ingredients in the evaluation data. The results are provided in Table 3, where “PPMI” uses $P_{a,b}$, “Co-occurrence” uses $D_{a,b}$, “spaCy” uses $S_{a,b}$, “Word2Vec” uses $W_{a,b}$, and “DIISH” uses ${\text{DIISH}}_{a,b}$ to rank substitutions. In the same table, “Poincaré” uses the cosine similarity between ingredients’ embeddings obtained from the Poincaré model trained over FoodOn classes to provide a comparison of our work to other recent embedding models. Note that results for The Cook’s Thesaurus’ data exclude the 100 target ingredients that were used as our development dataset.

**TABLE 3:** Evaluation results on our three datasets. Evaluation results on our three datasets. The top scores achieved for each dataset are highlighted in bold.

Ranking metric	MAP	MRR	RR at 5	RR at 10
Manually curated data				
Poincaré	0.215	0.282	0.394	0.437
PPMI	0.281	0.343	0.431	0.583
Co-occurrence	0.192	0.282	0.333	0.375
spaCy	0.274	0.371	0.500	0.653
Word2Vec	0.297	0.385	0.514	0.625
DIISH	**0.402**	**0.482**	**0.625**	**0.764**
The Cook’s Thesaurus data				
Poincaré	0.180	0.225	0.292	0.391
PPMI	0.178	0.225	0.291	0.377
Co-occurrence	0.175	0.252	0.331	0.414
spaCy	0.271	0.349	0.511	0.625
Word2Vec	0.266	0.358	0.503	0.617
DIISH	**0.381**	**0.448**	**0.622**	**0.725**
Food.com user review data				
Poincaré	0.125	0.196	0.290	0.374
PPMI	0.179	0.274	0.347	0.456
Co-occurrence	0.117	0.203	0.267	0.335
spaCy	0.167	0.269	0.405	0.524
Word2Vec	0.197	0.326	0.460	0.560
DIISH	**0.256**	**0.373**	**0.519**	**0.633**

We observe that DIISH consistently outperforms each of the other scoring metrics in all of our evaluation datasets. In constructing the DIISH, each similarity metric appears to capture slightly different aspects of good substitutions and contributes positively to improving the overall score. Additionally, applying our strategy to filter out ingredients that are super or subclasses of each other as not valid “substitutes” consistently improved results.

## 4 Discussion

From our results, we have observed that DIISH shows better performance at ranking ingredient substitutions than each of its components, which rely entirely on either explicit or implicit semantics. While the word embeddings are capable of capturing similarities between ingredient names and other associated words (e.g., spaCy’s embedding for “bake” is more similar to “potato” than to “garlic”), they cannot provide information such as food classification and capture some metrics of similarity rather than substitutability. Conversely, purely semantics-based methods are limited by the annotations captured in the knowledge graph. Relying only on explicitly annotated properties present in FoodKG would make our methods unable to leverage associations between ingredients and words relating to other aspects of cooking, such as preparation methods, utensils, or recipe names. Combining both explicit and implicit semantics in DIISH appears to provide some benefits of both methods, using some notion of similarity between word embeddings and applying intuitions to differentiate between “similar” and “substitutable” ingredients available through explicit semantics.

### 4.1 Use Case

Our approach shows promise when compared against our collected ground-truth substitution datasets. Additionally, the practical application of our ranked substitutions for particular people and recipes also demonstrate reasonable suggestions fitting for their dietary constraints. Let us consider a hypothetical diabetic patient who has a dietary goal of reducing the amount of carbohydrates in their meals. Table 4 shows some details about a recipe in FoodKG that this particular patient wants to make. Note that the recipe is for multiple servings.

**TABLE 4:** Example of a recipe in FoodKG.

Recipe: lemon and red onion roasted potatoes			
Ingredient	Quantity (calculated gram quantity)	Carbohydrates per 100 (g)	Total carbohydrates (g)
Black pepper	—	0	0
Coarse sea salt	—	0	0
Olive oil	6 tsp (26 g)	0	0
Lemon	2 (116 g)	9.3	10.8
Red onion	3 (480 g)	9.3	35.3
Potatoes	1 kg (1000 g)	17.5	175.0

Quantity is displayed as “—” where no quantity is specified in the recipe.

Nutritional information about the ingredients, linked from the USDA, can show the patient that potatoes are the greatest contributor to carbohydrates in this recipe (both in terms of its carbohydrates per 100 g and its quantity specified in the recipe). Since our example patient has a dietary goal of reducing their carbohydrate intake, our system can indicate to the patient that potatoes are the most “unhealthy” ingredient in the recipe and then display “healthy” potato substitutes that contain fewer carbohydrates. Table 5 shows the top five ranked substitutions for potatoes that match this constraint. Performing searches for low-carb potato substitutes on the web shows many similar options for good substitutes, supporting the utility of our substitute filtering and ranking approaches.

**TABLE 5:** Top five potato substitutions containing fewer carbohydrates than potatoes.

Target ingredient: potatoes	
**Ranked substitutes**	**Carbohydrates per 100 g**
1. Turnip	6.4 g
2. Squash	6.9 g
3. Cauliflower	5.0 g
4. Butternut squash	11.7 g
5. Zucchini	3.1 g

### 4.2 Limitations and Future Work

In our results, we can see some cases where our filtering strategy was unable to successfully remove undesirable options due to the class structure present in FoodOn and how FoodKG links ingredients to it. An example is provided in Table 6. We can see that the top five ranked options for fat were different varieties of fat (e.g., pork fat and duck fat). Many of these specific types of fats are lacking exact matches to appropriate ingredients in FoodOn. Additionally, due to how FoodOn classifies fats (“fat,” “oil,” and “fats and oils” are separate classes of products under “lipid food products”), our filtering strategy is unable to catch many of these results that negatively affect our substitution performance.

**TABLE 6:** Examples of substitutions that performed poorly against the ground-truth data.

Target	Ground-truth	DIISH’s top 5 ranked
Fat	Clarified butter	Low-fat milk
	Olive oil	Goose fat
	Vegetable oil	Bacon fat
	Cooking spray	Chicken fat
	…	Duck fat
Potato	Cauliflower	Baking potato
	Sweet potato	Sweet potato
	Rutabaga	Yukon gold potatoes
	Parsnip	Sweet dumpling squash
	…	Banana squash

Some errors also are caused by incorrect links between FoodKG ingredients and FoodOn classes. For example, the “baking potato” is linked to the food “potatoes gratine” in FoodOn because it has an alternate name of “potato bake,” which was the closest available match to baking potato. The variety of “Yukon Gold Potatoes” was not present in FoodOn, and thus, it was also linked to an incorrect food class. Such linking errors caused our substitution filtering strategy to fail, and this suggests a need for improvements to FoodKG’s linking procedure in the future (which was outside the scope of this work).

We also observe some limitations of our evaluation caused by our choice of ground-truth substitutions. While the sources of our evaluation datasets provided a wide variety of substitution options, it does not entirely cover all possible substitutions that people may use. For example, some websites consider varieties of squashes to be “good” substitutes for potatoes, but they did not appear as a substitute option in our evaluation data from The Cook’s Thesaurus.

Future work to more effectively utilize our presented approach will involve various improvements to FoodKG’s content. These improvements include improving linking between FoodKG ingredients, FoodOn classes, and the USDA’s nutritional information. Work also remains to improve FoodKG’s nutritional information calculation for individual ingredients in recipes. Although the USDA contains some information about common measurement units for ingredients, ingredient measurements in FoodKG’s recipes do not always have suitable matches with the USDA. Having more extensive coverage for automatic nutrition calculation will allow our ingredient substitution work to serve users better. We also may expand our evaluation datasets and devise a more comprehensive evaluation, such as considering more complex substitutions (i.e., one-to-many or many-to-one substitutions) and generating substitutions for specific contexts. Finally, work remains to integrate more aspects of user preference into the substitutions. Considering ingredient preference together with dietary constraints in the substitute ranking process will allow us to produce more personalized solutions.

### 4.3 Related Work

Previous studies applying computation to food and culinary arts have captured information such as flavor molecules, in FlavorDB (Garg et al., 2018), compounds and nutrients, in FooDB (The Metabolism Innovation Center, 2017), food categorization, in FoodOn (Dooley et al., 2018), or vector representations of foods, such as FoodEx2Vec (Eftimov et al., 2020). Other resources capturing recipes and associated information, such as RecipeDB (Batra et al., 2019), have also been developed. Efforts have been made to bring together the disparate sources of food information (Popovski. et al., 2019), but knowledge bases of food tend to either be limited in depth of information (as is the case for FoodKG (Haussmann et al., 2019)) or breadth of foods represented in the system.

Prior works to discover suitable substitution options have made limited use of semantic information. Achananuparp and Weber (2016) extracted food consumption patterns from MyFitnessPal’s food diaries, computing food item similarity based on the contexts in which food items appear. Akkoyunlu et al. (2017) similarly used the context of foods consumed together and applied a penalty to food items frequently consumed together. The use of NLP techniques and embedding similarity to search for substitute ingredients was explored by Pan et al. (2020), but this work lacked a formal evaluation of the generated substitutions. Our work differs from such previous works in that we leverage a greater degree of explicit semantic information about foods.

In works that leverage semantic information, ingredient substitutions are typically performed using explicit substitution options or rules. TAAABLE (Gaillard et al., 2015; Gaillard et al., 2017) performed recipe adaptations based on explicit rules and ingredient subclass information. Adaptations could also be used to perform queries for recipes based on what ingredients to include or exclude (Gaillard et al., 2014). Intellimeal (Skjold et al., 2017) performed recipe adaptations by searching for recipes that most closely matched a given user query and then performing modifications based on ingredient taxonomy similarity and substitution rules. These works differ from ours in which we do not rely only on explicit substitution rules, and our focus is to provide “healthy” substitutions based on user context.

More creative approaches to computational cooking have also been explored. Such works include the study by IBM’s Chef Watson (Varshney et al., 2019), which utilized big data approaches to generate novel recipes, by Evo Chef (Jabeen et al., 2019), which followed a semiautomated recipe generation with evolutionary algorithms, or by Calmon et al. (2020), which computed recipe similarity to uncover underlying patterns in ingredient usage. Such works often focus on the task of producing creative or novel recipes.

## 5 Conclusion

Managing and modifying nutritional intake from food is a meaningful way to maintain and improve personal health. Substituting ingredients is one way to help people improve their diets, but it can be difficult for people to identify viable substitutes for ingredients in a recipe and determine which substitutions are “healthier” for their particular dietary needs.

We develop a heuristic for ingredient substitutability, DIISH, by leveraging explicit and implicit semantic information about ingredients from various sources. Our heuristic can be used to automatically rank plausible substitution options. We additionally present an approach to navigate the search space of valid ingredient substitutions by utilizing nutritional information from the USDA and food classification from the FoodOn ontology. Based on constraints on nutritional content and classification, substitution options can be filtered to only rank substitutes that are “healthier” for a particular dietary goal. Although we focus on dietary constraints in this study, the approach could be used in a broader range of constraint types.

We evaluate our approach using three datasets of ingredient substitutions, curated from popular websites and user reviews. The scripts used to curate evaluation data as well as perform experiments are publicly available and open source^10^. We show that our new substitution ranking approach (DIISH) outperforms baseline methods, demonstrating the benefit of enriching embedding-based methods with domain-specific semantic information. Our results show promise toward empowering people to control their diets more effectively by automatically suggesting substitutions that conform to their dietary needs.

## Data Availability Statement

The raw data supporting the conclusions of this article will be made available by the authors, without undue reservation.

## Author Contributions

OS, CC, and DM conceptualized and supervised the project. SS conceptualized and implemented the methodology with support from OS and MG, curated evaluation data, and wrote the original manuscript draft. All authors contributed to revision of the manuscript and approved of the final submitted version.

## Funding

This work is partially supported by IBM Research AI through the AI Horizons Network.

## Conflict of Interest

Author C-HC was employed by the company IBM.

The authors declare that this study received funding from IBM.

## Footnotes

1
https://www.allrecipes.com

2
https://www.food.com

3
http://www.allrecipes.com/article/common-ingredient-substitutions

4
https://catalog.data.gov/dataset/food-and-nutrient-database-for-dietary-studies-fndds

5
http://www.allrecipes.com/article/common-ingredient-substitutions

6
http://www.foodnetwork.ca/kitchen-basics/photos/18-most-common-ingredient-substitutions

7
http://extension.colostate.edu/docs/pubs/foodnut/09329.pdf

8
http://www.ag.ndsu.edu/publications/food-nutrition/ingredient-substitutions/fn198.pdf

9
http://foodsubs.com/

10
https://foodkg.github.io/subs.html
